# Heterogeneity of Cardiomyocyte Lipid Droplets and Their Pathophysiological Implications in Cardiovascular Diseases

**DOI:** 10.3390/cells15181648

**Published:** 2026-09-11

**Authors:** Xinjuan Lei, Wei Zhang

**Affiliations:** 1 Laboratory of Cell Biology, Genetics and Developmental Biology, Shaanxi Normal University College of Life Sciences, Xi’an 710062, China; xinjuanl@snnu.edu.cn; 2 Institute of Pharmacological Medicine, Jiangxi Academy of Medical Sciences, Nanchang 330006, China

**Keywords:** cardiomyocyte lipid droplet, lipid droplet heterogeneity, cardiac lipid metabolism, lipotoxicity, mitochondrial coupling

## Abstract

**Highlights:**

**What are the main findings?**

- A conceptual framework is proposed for cardiomyocyte lipid droplet (CLD) heterogeneity, classifying distinct CLD subpopulations along three dimensions: morphology, organelle connectivity, and molecular composition.
- Mitochondrion-associated CLDs represent a functionally specialized subpopulation whose coupling to mitochondria—mediated primarily by PLIN5—supports efficient fatty acid oxidation and myocardial metabolic flexibility; disruption of this coupling is linked to lipotoxicity and cardiac dysfunction.

**What are the implications of the main findings?**

- Dysregulation of distinct CLD subpopulations may contribute to the pathogenesis of cardiovascular diseases, including heart failure and diabetic cardiomyopathy.
- Targeting specific CLD subpopulations may represent a potential therapeutic strategy, although direct validation at the single-droplet level has yet to be achieved.

**Abstract:**

The adult heart relies on fatty acid β-oxidation for energy, and cardiomyocyte lipid droplets (CLDs) are central to myocardial lipid homeostasis. CLDs are now recognized to protect cardiomyocytes against lipotoxicity, endoplasmic reticulum (ER) stress, oxidative stress, and nutrient deprivation. Moreover, accumulating evidence suggests that CLDs within the same cardiomyocyte are not uniform but may exhibit heterogeneity in size, subcellular localization, protein composition, and metabolism. Here, we propose a conceptual framework that classifies CLD heterogeneity along three dimensions: morphology, organelle connectivity, and molecular composition. We first define the biological characteristics of CLDs and categorize their heterogeneity. We then discuss the molecular pathways maintaining this heterogeneity, their roles in the healthy heart, and their dysregulation in cardiovascular diseases. Finally, we review preclinical strategies targeting CLD heterogeneity and outline challenges and future directions for clinical translation. This framework integrates current evidence while acknowledging that direct molecular validation awaits future single-droplet omics technologies.

## 1. Introduction

Cardiovascular diseases remain the leading cause of morbidity and mortality worldwide, imposing a massive global public health burden [1]. The heart is the organ with the greatest energy demand in the human body and requires a continuous supply of ATP to support rhythmic contractions throughout the lifespan. Under physiological conditions, 60–90% of the ATP produced in the adult heart is derived from mitochondrial fatty acid β-oxidation, highlighting myocardial lipid homeostasis as a critical factor for maintaining normal cardiac function [2]. Dysregulation of myocardial lipid metabolism is a universal hallmark of nearly all cardiovascular diseases, including diabetic cardiomyopathy, myocardial infarction and heart failure, and is closely associated with adverse clinical outcomes [3]. Therefore, elucidating the regulatory mechanisms of myocardial lipid homeostasis is critical for identifying novel therapeutic targets for cardiovascular diseases.

Lipid droplets (LDs) are evolutionarily conserved organelles consisting of a neutral lipid core enclosed by a phospholipid monolayer decorated with LD-associated proteins (LDsAPs). For decades, LDs have been regarded as inert, passive intracellular depots for excess lipid storage, with no active biological functions beyond energy reserves. However, this traditional view has been fundamentally revolutionized in the past two decades. Accumulating evidence from cell biology, metabolic research, and cardiovascular studies has established that LDs are highly dynamic, multifunctional organelles involved in a wide range of cellular processes, including energy metabolism, signal transduction, organelle crosstalk, protein quality control, and stress adaptation. In the heart, LDs are particularly critical, as they act as the central hub linking fatty acid uptake, storage, and utilization for energy production, while also protecting cardiomyocytes from lipotoxic injury caused by excess free fatty acids [4].

In this review, we propose a conceptual framework that defines and classifies CLD heterogeneity along three dimensions: morphology, organelle connectivity, and molecular composition of CLDs. This framework is built upon converging lines of preclinical evidence; however, we emphasize that direct molecular validation at the single-droplet level within individual cardiomyocytes remains to be achieved. In the following sections, we first outline the unique biological characteristics of CLDs and the rationale for their classification into multiple dimensions. We then examine the molecular regulatory networks that maintain distinct CLD subpopulations and discuss their specialized physiological functions in the healthy heart. Next, we explored how dysregulation of CLD heterogeneity may contribute to the pathogenesis of major cardiovascular diseases. Finally, we review preclinical strategies for targeting CLD heterogeneity for cardiovascular protection, identify key obstacles to clinical translation, and highlight the emerging technologies and future research priorities. By integrating current evidence into a coherent framework, this review aims to advance the understanding of myocardial lipid biology and stimulate novel approaches for the treatment of cardiovascular diseases.

## 2. Conceptualizing Cardiomyocyte Lipid Droplet Heterogeneity: Classification and Methodology

In contrast to the historical view that LDs are merely inert energy storage depots, distinct types of LDs exist across different tissues, cell types, and even within individual cells [5]. LD heterogeneity has been increasingly regarded as a key regulator of metabolic processes, with distinct LD subpopulations likely serving different functions [6]. The heart predominantly relies on fatty acids for energy production; therefore, CLDs play a critical role in cardiac physiology [2]. Although LD heterogeneity has received considerable attention in other tissues, and multiple independent studies in the heart have reported the presence of different types of LDs, a comprehensive discussion of this phenomenon in cardiomyocytes is still lacking. Lipid droplets in cardiomyocytes differ from those in white adipocytes, exhibiting a small and dispersed distribution pattern. Through transmission electron microscopy, immunofluorescence, and proteomic analyses, researchers have identified distinct LD populations in the heart that differ in size, subcellular localization, and molecular composition [6,7,8,9,10]. These observations support the concept that individual cardiomyocytes may harbor functionally distinct LD subpopulations, a concept we term cardiomyocyte lipid droplet (CLD) heterogeneity. Here, we synthesize the evidence that has led us to propose the concept of CLD heterogeneity and discuss its potential relevance to cardiovascular disease. A framework for understanding CLD heterogeneity has been proposed by Henne and Cohen (2026), who described LD diversity from three perspectives: compositional heterogeneity, differences in size and spatial organization, and the diverse contacts LDs form with other organelles [5]. In the cardiac context, these perspectives can be operationalized to provisionally recognize distinct CLD subpopulations when particular combinations of size, molecular decoration, and organelle association recur together. For example, CLDs enriched in perilipin 5 (PLIN5, encoded by *Plin5*) are frequently found adjacent to mitochondria and are thought to participate in fatty acid channeling, whereas those with reduced PLIN5 and increased hormone-sensitive lipase (HSL) appear to be more lipolytically active [7,11]. Similarly, CLDs in healthy hearts are predominantly smaller than 0.5 μm, whereas under pathological lipid loading they can range from approximately 0.3 to 1.9 μm [7]. These recurrent combinations—rather than any single feature—may provide a basis for distinguishing one provisional subpopulation from another, although direct experimental validation at the single-droplet level remains to be achieved. The operational criteria are summarized in Table 1. By providing a unified perspective on these previously scattered observations, we aim to offer a conceptual foundation for future investigations into how distinct CLD subpopulations contribute to cardiac physiology and pathogenesis.

### 2.1. Unique Biological Characteristics of Cardiomyocyte Lipid Droplets

The distinctive biological characteristics of CLDs are consistent with the physiological functions of the heart. In cardiomyocytes, lipid droplets generally range in size from 0.1 μm to 2 μm, with an average diameter of less than 1 μm under normal physiological conditions [8]. This contrasts with lipid droplets that constitute the majority of the volume in white adipocytes [7]. The dispersed distribution of lipid droplets within cardiomyocytes is not random; rather, it represents an adaptive feature that facilitates efficient energy transfer through interactions with various organelles [12].

In 2024, Hu et al. conducted a study in which lipid droplets (LDs) were stained with Bodipy 493/503, and mitochondria were labeled using MitoTracker Red in primary mouse cardiomyocytes. The researchers quantified the proportion of lipid droplets in direct contact with mitochondria using confocal fluorescence microscopy. Direct contact was defined as the absence of visible gaps between the two organelles. In primary mouse cardiomyocytes, quantitative fluorescence imaging revealed that more than 70% of CLDs were in direct contact with mitochondria under basal conditions. A comparably high proportion of LD–mitochondrial contact has also been observed in H9C2 cardiomyoblasts [13]. Additionally, corroborative statistical data obtained through transmission electron microscopy in the same study supported these results. This distinctive structural feature is more pronounced in cardiomyocytes than in other cell types. These contact sites facilitate the direct entry of fatty acids released from CLDs into the mitochondria for β-oxidation, eliminating the need for extensive diffusion through the cytoplasm. This unique arrangement optimizes energy supply efficiency to sustain continuous cardiac contractions while preventing the accumulation of harmful free fatty acids in the cytoplasm, a mechanism that may help protect cardiomyocytes against lipid-induced toxicity [14,15].

A crucial characteristic of CLDs is their tissue-specific protein expression. In the liver and adipocytes, perilipin 1 (PLIN1), PLIN2, and perilipin 3 (PLIN3) are predominantly found on the surface of LDs, whereas high levels of PLIN5 expression are detected in CLDs [16,17,18,19]. PLIN5 plays a key role in facilitating CLD–mitochondrial interactions and promoting cardiac lipid catabolism [19]. This distinctive protein expression pattern in different tissues influences the specific functional attributes of CLDs, emphasizing rapid and effective energy provision while preserving the lipid balance in cardiac tissue.

### 2.2. Multidimensional Classification of Cardiomyocyte Lipid Droplet Heterogeneity

CLD heterogeneity represents a tightly regulated, multilayered biological phenomenon, rather than a random morphological variation. Among the three interrelated dimensions we propose, the most intuitive and well-characterized are size and number heterogeneities. Under normal physiological conditions, cardiomyocytes maintain a delicate equilibrium between lipid uptake and oxidation, ensuring the stability of the lipid droplet content and morphology within the cells. However, pathological conditions can disrupt this balance, which is associated with alterations in the quantity and size of lipid droplets in cardiomyocytes. A study in 2018 demonstrated that high-fat diet induction could increase the lipid droplet content in the myocardium of mice [20]. Subsequent research by Latchman’s team in 2025 revealed that dilated cardiomyopathy, ischemic cardiomyopathy, and hypertrophic cardiomyopathy in a pathological milieu could decrease the number of large lipid droplets in cardiomyocytes [8]. Reticulon 3 (RTN3, encoded by *Rtn3*) plays a role in facilitating the targeted transport of fatty acids to the ER. Its overexpression is linked to impaired systolic and diastolic heart functions. In this pathological context, RTN3 overexpression increases both the quantity and size of lipid droplets in cardiomyocytes, with a notable increase in the proportion of large-diameter lipid droplets [21].

In addition to pathological influences, lipid droplets within cardiomyocytes exhibit morphological heterogeneity under basal physiological conditions. Latchman et al. performed ultrastructural analysis based on transmission electron microscopy (TEM) of human left ventricular tissue and quantified the perimeter, feret diameter, and circularity of LDs in cardiomyocytes, confirming the presence of LDs of diverse sizes and shapes within the myocardium [8]. Similarly, using TEM and fluorescence staining of mouse heart tissue, Son et al. reported that LDs isolated from the heart are composed of subpopulations with distinct size ranges, suggesting that cardiomyocytes vary in LD size [7]. Guo et al. further analyzed the size distribution of LDs in mouse left ventricular tissue using TEM and reported that the diameter of LDs in cardiomyocytes was not uniform but spanned a broad range, from less than 400 nm to greater than 800 nm [21]. Collectively, these observations indicate the existence of LD subpopulations of different sizes within cardiomyocytes. However, direct evidence at the single cardiomyocyte level remains limited, and further studies are needed to validate this concept.

LDs in different subcellular compartments interact with specific organelles, microenvironments and energy demands. In adult cardiomyocytes, the majority of CLDs are closely associated with mitochondria [13]. Electron microscopy of the left ventricular muscle of fasted mice revealed that this proportion can reach 90% [22]. This close proximity between CLDs and mitochondria enhances lipid oxidation efficiency. In cardiomyocytes, the spatial relationship between CLDs and the mitochondria is tightly regulated. PLIN5 is involved in protein expression at the outer mitochondrial membrane and promotes the binding of mitochondria to CLDs. Using confocal microscopy, electron microscopy, and Western blotting in neonatal cardiac myocytes, Wang et al. demonstrated that PLIN5 on the CLD surface recruits mitochondria via its C-terminal domain [22]. Overexpression of PLIN5 in the heart not only increases the number of CLDs but also enhances the connection between mitochondria and CLDs, which may alleviate the cardiac damage caused by lipotoxicity [23]. In addition to PLIN5, heat shock cognate 70 (HSC70), a heat shock protein localized on CLDs, interacts with the 649–692 amino acid domain of mitofusin 2 (MFN2) on the outer mitochondrial membrane. This interaction facilitates membrane contact between CLDs and mitochondria, thereby promoting fatty acid oxidation [13].

Myocardial lipid droplets can also form membrane contact sites with organelles such as the ER and lysosomes. The ER is involved in CLD formation, and a continuous membrane structure has been observed between the two. This contact is mediated by seipin and fat storage-inducing transmembrane (FIT) proteins. The ER plays a role in buffering free fatty acids by regulating lipid droplet formation, thereby potentially protecting cells and reducing lipid toxicity and ER stress. Material is exchanged between CLDs and lysosomes. Lysosomes facilitate the release of fatty acids for reuse, thereby preventing excessive lipid accumulation. In cardiomyocytes, lipid droplets form contact sites among the ER, mitochondria, and lysosomes and may regulate the survival, autophagy, and hypertrophic fibrosis processes in cardiomyocytes through the ROS oxidative stress axis and the PLIN5-PPARα-PI3K/AKT signaling axis [14]. Additional LD-associated proteins that regulate LD positioning and facilitate organelle interactions have been identified in other tissues. However, the functional roles of these proteins in cardiomyocytes have not been extensively studied, and their potential contribution to the heterogeneity of CLDs represents a significant area for future research [24,25].

Another source of CLD heterogeneity lies in the variation in surface protein and lipid core composition, which provides a molecular basis for the morphological and functional diversity of lipid droplets. As early as 2006, Yamaguchi et al. proposed that the surface protein composition of LDs changes in response to cellular physiological states [26]. In 2016, Li et al. performed iTRAQ-based quantitative proteomic analysis of lipid droplets isolated from rat hearts and identified a complex proteome comprising more than 752 associated proteins, including classic members of the perilipin family, such as PLIN2, PLIN3, and PLIN5 [27]. In 2025, a study directly demonstrated the presence of compositionally distinct CLDs in cardiomyocytes. By combining immunofluorescence of cardiac tissue sections, transmission electron microscopy of cardiomyocytes, and proteomic analysis of isolated lipid droplets, the authors reported that the abundance of proteins, such as PLIN5 and HSL, on cardiac LDs differed across mouse models. Furthermore, TEM directly captured distinct metabolic modes, including lipolysis and lipophagy [28]. Together, this multilayered evidence supports the conclusion that lipid droplets in mouse cardiomyocytes are heterogeneous in terms of both surface protein composition and metabolism, and that these differences may contribute to their functional heterogeneity under physiological and pathological conditions.

In addition to the differences in membrane proteins under basal conditions, the protein composition on the surface of lipid droplets is altered in response to various pathological states. For instance, in pressure overload-induced dysfunctional rat hearts, 27 proteins on lipid droplets increased, whereas 16 proteins decreased [27]. In the lipid-toxic mouse model MHC-*Pparg1*, 3296 LDAPs were identified, of which 320 were upregulated and 110 were downregulated compared with those in the control group. The expression of perilipin (PLIN) family proteins decreased uniformly [7]. These findings suggest that the protein composition of LDsAPs on CLDs may be heterogeneous and that there is a close association between LDsAPs and the function, localization, and physiological state of CLDs, indicating that LDsAPs undergo remodeling in response to changes in the cellular environment. However, it should be noted that current proteomic and lipidomic data on cardiac LDs have largely been obtained from bulk cardiac tissue homogenates or pooled LD fractions rather than from single cardiomyocyte or single LD analyses. Therefore, the molecular heterogeneity of LDsAPs across individual CLDs within a single cardiomyocyte remains to be directly validated. Future studies employing single cardiomyocyte proteomics or single-LD proteomic approaches, combined with spatial imaging at subcellular resolution, will be essential to definitively establish whether distinct CLD subpopulations possess characteristic LDAP profiles and lipid compositions.

In addition to LDsAPs, the lipid composition of CLDs contributes to their heterogeneity. Multiple studies have highlighted the role of lipid composition in defining distinct LD subpopulations across different cell types [5,6]. In a lipid toxicity mouse model, a 2% reduction in the core lipid content was observed in cardiac LDs, suggesting that the proportions of core lipids and monolayer phospholipids in CLDs may vary depending on the cellular milieu and that CLDs with distinct lipid compositions may exist in cardiomyocytes. However, similar to current proteomic data, lipidomic evidence has been obtained primarily from bulk cardiac tissue or pooled LD fractions rather than from single cardiomyocyte or single-LD analyses. Therefore, the lipid heterogeneity of individual CLDs remains to be directly validated by future single-cell lipidomics or single-LD lipid profiling technologies [7].

### 2.3. Key Technologies for Characterizing Cardiomyocyte Lipid Droplet Heterogeneity

Advances in our understanding of CLD heterogeneity have been closely linked to technological innovations in the past two decades. TEM remains the gold standard for visualizing the ultrastructure of CLDs, enabling the observation of LD size and nanoscale CLD–mitochondrial contact sites. Confocal laser scanning microscopy is the most widely used technique for the quantitative analysis of CLD number, size, and subcellular distribution in both fixed cells and tissue sections, owing to its high throughput and compatibility with multichannel fluorescence labeling. Super-resolution fluorescence microscopy techniques, such as STED, SIM, and STORM, have overcome the optical diffraction limit and may, in the future, enable the visualization of micro-CLDs and the spatial distribution of CLD-associated proteins at the nanoscale in living cells [7,20].

In terms of molecular characterization, density gradient centrifugation combined with bulk proteomics and lipidomics has provided foundational data on the molecular composition of CLDs. These bulk approaches have identified hundreds of LD-associated proteins and revealed condition-dependent remodeling of the cardiac LD proteome. However, because bulk analyses cannot distinguish the protein profiles of individual CLDs within the same cardiomyocyte, the molecular heterogeneity of distinct CLD subpopulations at the proteomic level remains unknown. The recent development of single-LD proteomics and lipidomics in other cell types offers a promising avenue for directly characterizing the molecular heterogeneity of individual CLDs in the heart, although such studies have yet to be performed in cardiomyocytes [7].

In addition to the methods mentioned above, several emerging technologies developed in other systems enable the separation or visualization of distinct LD subpopulations, and some of them may be adapted to cardiac research in the future. For instance, multistep fractionation protocols separate LD subpopulations based on factors such as size or organelle association, thereby identifying subpopulations with structural and functional differences [5]. These methods rely on the intrinsic buoyancy of LDs in aqueous buffers, but they typically require large amounts of starting material and may disrupt labile organelle contacts. Immunofluorescence microscopy has been used to label specific LD subpopulations defined by surface proteins, such as perilipins [5]; the development of additional markers may therefore represent a useful direction for future cardiac LD research. Polarized light microscopy enables direct visualization of liquid–crystalline-phase LDs and can be combined with vital dyes [5]. Cryo-electron microscopy and three-dimensional electron tomography provide high-resolution views of LD ultrastructure [5]. In addition, organelle contact sensors—including split fluorescent protein systems, Contact-FP, and FABCON—have been used in other cell types to visualize dynamic LD–organelle contacts [5]. The adaptation of these technologies to cardiomyocytes, together with spatial lipidomics and single-LD proteomic approaches, may help to test the concept of CLD heterogeneity and enable direct experimental validation in the future.

**Figure 1:**
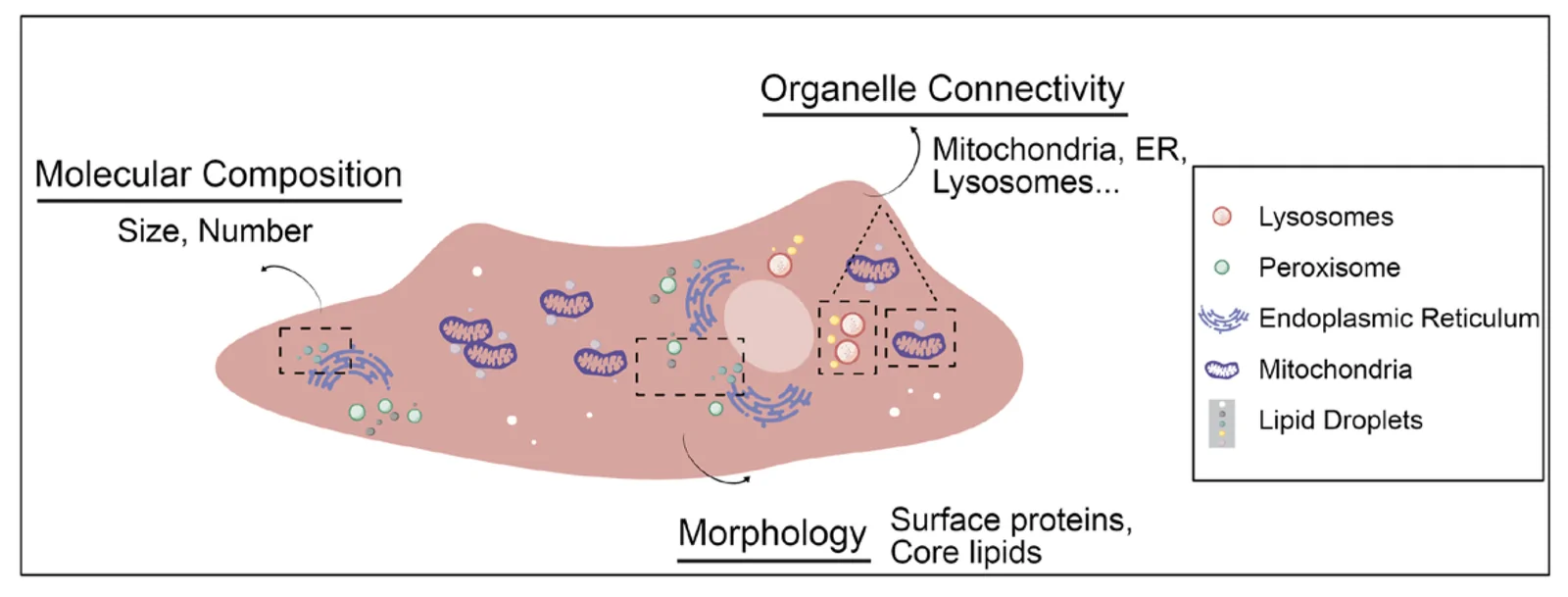
Proposed conceptual framework of cardiomyocyte lipid droplet heterogeneity. Schematic illustration of a single cardiomyocyte showing the proposed coexistence of distinct CLD subpopulations. Size heterogeneity is represented by differences in droplet size, compositional heterogeneity is indicated by different colors, and organelle connectivity is illustrated by the proximity of individual CLDs to mitochondria, the endoplasmic reticulum, lysosomes, or peroxisomes. Lipid droplets outlined with dashed lines indicate representative patterns of the three classification dimensions, with arrows linking each representative droplet to the corresponding surrounding panel. Created with BioGDP.com (https://BioGDP.com) [29].

In summary, CLD heterogeneity is a multidimensional, tightly regulated biological phenomenon intimately linked to the functional diversity of lipid droplets. In this section, we outline the biological characteristics of CLDs. An overview of the proposed conceptual framework for CLD heterogeneity is shown in Figure 1. While current approaches, including electron microscopy, immunofluorescence, and density gradient centrifugation, have provided evidence for the existence of distinct types of LDs in the heart, direct validation of this concept at the single cardiomyocyte or single droplet level remains lacking. Future studies employing single-LD proteomics, single-LD lipidomics, and functional characterization of individual lipid droplets within intact cardiomyocytes will be essential to substantiate the proposed framework of CLD heterogeneity. Given that LDs have been implicated in oxidative stress, inflammatory responses, and autophagy in cardiomyocytes [4], further investigation in this area may help elucidate the pathophysiological roles of CLDs in cardiovascular disease.

## 3. Molecular Mechanisms Underlying the Proposed Framework of CLD Heterogeneity

In the previous section, we outlined the multidimensional heterogeneity of CLDs and proposed a conceptual framework that classifies CLDs into distinct subpopulations. If this framework holds, the observed diversity of CLDs is unlikely to reflect random or passive variation in lipid storage, but instead implies the existence of a dynamic, multilayered regulatory network that actively maintains distinct CLD subpopulations. Under physiological conditions, such a network is expected to preserve the balance of different CLD subpopulations to meet the fluctuating energy demands of the beating heart. Under pathological stress, dysregulation of CLDs may underlie the disruption of CLD homeostasis, potentially contributing to myocardial metabolic disorders and cardiac dysfunction. In this chapter, we examine the core molecular mechanisms that may govern CLD heterogeneity within this conceptual framework, focusing on the regulatory roles of lipid droplet-associated proteins, shaping of spatial and functional heterogeneity by organelle crosstalk, and the upstream influence of transcriptional, epigenetic, and microenvironmental cues.

### 3.1. Lipid Droplet-Associated Proteins: Core Molecular Switches of CLD Heterogeneity

A 2026 review revealed that the diversity of lipid droplets, including the heterogeneity of their protein composition, contributes to their distinct functions [5]. Consistent with this, in this review, we propose that lipid droplet-associated proteins anchored to the phospholipid monolayer of CLDs may play a crucial role in regulating CLD heterogeneity. The PLIN family is the major family of LD surface proteins and exhibits tissue-specific expression patterns that contribute significantly to the distinct characteristics of CLDs. Among the PLIN family members, PLIN5 has emerged as a pivotal isoform in the adult heart and plays a central role in maintaining the characteristics of mature and functional CLD subpopulations in the adult heart. Through direct interactions with mitochondrial membrane proteins and lipolytic enzymes, PLIN5 not only enhances the physical association between CLDs and mitochondria but also, depending on its PKA phosphorylation status, restricts or facilitates the lipolytic activity of CLDs to flexibly meet the energy demands of cardiomyocytes. The abundance and subcellular localization of PLIN5 are thought to influence the proportion of mature functional CLDs, the efficacy of fatty acid transport, and the preservation of metabolic adaptability in cardiac tissue [22,30].

In contrast to PLIN5, other perilipin isoforms may contribute to the regulation of distinct CLD subpopulations, although direct evidence in cardiomyocytes remains limited. PLIN3 has been implicated in LD biogenesis in several noncardiac cell types, where it is recruited to the ER during LD formation and promotes the generation of nascent LDs [31]; however, its specific role in CLD subpopulations has not been investigated. Perilipin 2 (PLIN2; encoded by *Plin2*) is the predominant LD-coat protein in the heart under basal conditions. Cardiac-specific overexpression of *Plin2* in mice leads to the accumulation of small LDs in association with mitochondrial chains in atrial cardiomyocytes, resulting in increased atrial TAG content and connexin 43 (Cx43) remodeling without adverse effects on ventricular function [32]. These findings suggest that PLIN2 primarily stabilizes LDs and regulates lipid storage in cardiomyocytes. However, whether PLIN2 defines a functionally distinct CLD subpopulation under physiological or pathological conditions remains unclear.

In addition to the PLIN family, other LD-associated proteins may contribute to CLD heterogeneity by promoting either lipid synthesis or degradation on LD surfaces, depending on their specific roles. Diacylglycerol acyltransferases (DGAT1 and DGAT2), the key enzymes that catalyze the final step of triglyceride synthesis, are located on the ER and cardiac LDs, where they regulate CLD biogenesis, growth, and size by controlling the rate of triglyceride synthesis [7]. Conversely, adipose triglyceride lipase (ATGL), the rate-limiting enzyme for triglyceride hydrolysis, is associated with cardiac LDs. Quantitative proteomic analysis of LDs isolated from rat hearts revealed that ATGL levels in pressure overload-induced dysfunctional hearts are significantly decreased compared with those in normal hearts [27,33], suggesting that altered ATGL localization may contribute to the excessive lipid accumulation observed in patients with cardiac dysfunction. Together, the coordinated actions of lipogenic and lipolytic enzymes on the LD surface may influence the metabolic fate of individual CLDs, and their dysregulation could shift the balance between distinct CLD subpopulations, thereby contributing to the pathogenesis of cardiac diseases.

### 3.2. Organelle Crosstalk Shapes the Spatial and Functional Heterogeneity of CLDs

Subcellular localization is a key factor in the functional specificity of CLDs, and the spatial distribution and functional differentiation of distinct CLD subpopulations are largely shaped by direct physical and functional crosstalk between CLDs and other organelles. This organelle interaction is a central mechanism underlying the spatial and functional heterogeneity of CLDs in cardiomyocytes.

Crosstalk between LDs and the ER is a fundamental source of CLD heterogeneity in hepatocytes. All LDs originate from the ER membrane, and the local lipid synthesis capacity, protein recruitment efficiency, and membrane curvature of the ER play critical roles in determining the size, number, and initial molecular composition of nascent LDs. ER-associated nascent LDs can either remain attached to the ER for continuous growth and maturation or bud off into the cytosol for translocation to other subcellular compartments. This difference in fate represents an early step in the formation of LD localization heterogeneity, which also underlies the spatial heterogeneity of CLDs in cardiomyocytes [34,35].

The interaction between CLDs and mitochondria is the primary and functionally critical interplay in cardiomyocytes. Most CLDs in healthy cardiomyocytes establish stable membrane contact sites with mitochondria [13], facilitating the direct and efficient transfer of fatty acids from CLDs to mitochondria for β-oxidation. This process may limit the cytoplasmic diffusion of free fatty acids, thereby reducing the risk of lipid toxicity. The extent of the CLD–mitochondrial association influences the functional status of individual CLDs and serves as a key indicator for distinguishing energy-supplying functional CLDs from other subgroups [8,23,36,37].

Although both the PLIN5- fatty acid transport protein 4 (FATP4) and MFN2-HSC70 complexes have been shown to mediate LD–mitochondria contacts, the strength of evidence supporting their roles in the heart differs considerably. The MFN2-HSC70 complex directly mediates LD–mitochondrion tethering in cardiomyocytes, providing both structural and functional evidence for its role in cardiac mitochondrial lipid contact (MLC) formation [13]. In contrast, although PLIN5 is well established as a functional mediator of CLD–mitochondria coupling in the heart, its interaction with FATP4 has only been characterized in noncardiac cell lines. Direct evidence for a PLIN5-FATP4 tethering complex at the CLD–mitochondria interface in cardiomyocytes is currently lacking [38]. Other proteins proposed to mediate LD–mitochondrion contacts in other tissues, including synaptosome-associated protein 23 (SNAP23) and mitoguardin 2 (MIGA2), have been implicated in LD–mitochondrion tethering in adipocytes and hepatocytes, but have not yet been studied in the heart [39,40]. A systematic evaluation of the molecular architecture of CLD–organelle contact sites in cardiomyocytes, distinguishing between functional mediators and structural tethers, will be important in future research.

Peroxisomes are another organelle whose interaction with CLDs may have functional implications, although this possibility has not been explored in the heart. Peroxisomes are essential for the β-oxidation of very long-chain fatty acids, which cannot be directly metabolized by mitochondria and must first undergo chain shortening in peroxisomes before being transferred to mitochondria for complete oxidation. In hepatocytes, LDs supply fatty acids to peroxisomes through direct organelle contact, and this LD–peroxisome interaction is thought to facilitate the channeling of very long-chain fatty acids into peroxisomal β-oxidation. However, it remains unclear whether a subpopulation of CLDs specifically associates with peroxisomes in cardiomyocytes and whether peroxisome–CLD contacts contribute to the handling of very-long-chain fatty acids in cardiac tissue. Given the high metabolic demand of the heart, the existence of peroxisome-associated CLDs represents an intriguing possibility that may constitute an additional yet unrecognized axis of CLD heterogeneity.

The concept that the subcellular localization of LDs is coupled to their metabolic function has been well established in skeletal muscle, where LDs are classified into two spatially and functionally distinct populations: intermyofibrillar LDs, located between myofibrils in close proximity to mitochondria and serving primarily as local energy reservoirs for contraction, and subsarcolemmal LDs, positioned beneath the sarcolemma and thought to participate in membrane lipid metabolism and signaling [41]. Whether similar spatial compartmentalization occurs in cardiomyocytes remains unknown. However, given the structural and metabolic similarities between cardiac and skeletal muscles, including their shared dependence on fatty acid oxidation, highly organized myofibrillar architecture, and comparable distribution patterns of mitochondria, CLD subpopulations in cardiomyocytes may also be spatially organized according to their metabolic functions. If such spatial compartmentalization exists in the heart, it would add an important dimension to the concept of CLD heterogeneity proposed in this review, linking subcellular positioning directly to functional specialization in cardiomyocytes. Systematic ultrastructural analyses of CLD distribution in relation to subcellular landmarks in cardiomyocytes, analogous to those performed in skeletal muscle, are needed to explore this possibility. In addition to the mitochondria and ER, the interplay between CLDs and other organelles may further shape this spatial landscape.

Additionally, crosstalk between CLDs and other organelles may contribute to CLD heterogeneity. CLDs have been shown to be associated with lysosomes, and lysosome-mediated lipophagy involving chaperone-mediated autophagy, which involves the degradation of LD-associated proteins such as PLIN2 and potentially PLIN3, has been proposed as a mechanism for LD component degradation [8,42]. Whether this process contributes to the quality control of CLD subpopulations or selectively targets damaged and dysfunctional CLDs in cardiomyocytes remains to be determined.

### 3.3. Upstream Transcriptional, Epigenetic and Microenvironmental Modulation

The core regulatory networks of LDsAPs and organelle crosstalk are thought to be further shaped by upstream transcriptional, epigenetic, and microenvironmental cues, which may serve as key mediators of the dynamic remodeling of CLD heterogeneity in response to physiological and pathological changes. Epigenetic modifications may also contribute to the regulation of CLD. For instance, methyl donor deficiency has been shown to alter the methylation and acetylation status of peroxisome proliferator-activated receptor γ coactivator 1-alpha (PGC-1α), leading to disrupted fatty acid oxidation and lipid accumulation in the myocardium [43]. In addition, non-coding RNAs have been implicated in LD biology: LIPTER, a cardiomyocyte-enriched lncRNA, directly participates in LD transport by binding phosphatidic acid and phosphatidylinositol 4-phosphate on the LD surface, as well as the myosin heavy chain 10 (MYH10) motor protein, thereby connecting LDs to the cytoskeleton [12], suggesting that LIPTER-mediated LD transport may contribute to the regulation of the spatial positioning of CLDs within cardiomyocytes. In addition, specific miRNAs have been reported to regulate PLIN5 expression in the heart; for instance, miR-370 and miR-292-5p protect the myocardium during ischemia/reperfusion injury by activating *Plin5* [44], suggesting that miRNA-mediated regulation of LD-associated proteins may influence the function of CLDs. Environmental factors, such as extracellular fatty acid availability, oxygen levels, cardiac workload, and hormonal signals, including insulin and adrenergic stimulation, may influence the composition and distribution of CLD subpopulations [4]. For instance, fasting or exercise training activates the PPARα–PGC-1α pathway, increasing the number of mature functional CLDs and enhancing the association between CLDs and mitochondria. Conversely, prolonged exposure to high levels of fatty acids, which is common in conditions such as diabetes and obesity, is associated with CLD accumulation and impaired differentiation of functional CLDs [7,20,45,46].

In summary, CLD heterogeneity may be governed by a multilayered regulatory network, with LDsAPs as central molecular mediators, organelle crosstalk as spatial and functional organizers, and transcriptional, epigenetic, and microenvironmental cues as upstream dynamic regulators. If operative, such a network would be expected to preserve the balance of different CLD subpopulations in a healthy heart, and its disruption may be linked to the progression of cardiac dysfunction. In the next section, we examine the proposed functional roles of heterogeneous CLD subpopulations in cardiac health and disease.

## 4. Functional Roles of CLD Subpopulations in Health and Disease

In the preceding chapter, we examined the molecular mechanisms that may govern the formation and maintenance of CLD heterogeneity within a conceptual framework of CLD development. If this framework holds, the differentiation of distinct CLD subpopulations would not merely represent random variation in lipid storage but may reflect an adaptive mechanism that matches the unique physiological demands of the adult heart—continuous rhythmic contraction with fluctuating energy requirements coupled with the need to avoid lipotoxic injury from excess free fatty acids. Under this model, each CLD subpopulation—defined by its size, subcellular localization, and molecular profile—could perform specialized physiological functions that collectively contribute to myocardial metabolic homeostasis, contractile function, and stress resistance. In this chapter, we discuss the proposed physiological roles of distinct CLD subpopulations in the healthy heart and examine how CLD heterogeneity may be dynamically remodeled in response to physiological stimuli.

### 4.1. Mitochondrially Associated CLDs: Functional Roles and PLIN-Mediated Regulation

The most extensively studied physiological function of CLDs is to serve as an immediate energy reservoir for cardiac muscle contraction, a role primarily attributed to the mature and functionally active subset of CLDs. These CLDs, often referred to as mitochondrial lipid droplets, are characterized by their close association with mitochondria. Most CLDs in healthy cardiomyocytes establish stable membrane contact sites with mitochondria [13], and PLIN5 is considered a key mediator of this physical association. Unlike the systemic energy storage function of LDs in adipocytes, these mitochondrially associated CLDs are thought to facilitate the efficient channeling of fatty acids released during lipolysis directly to the mitochondria for β-oxidation, a process that may limit prolonged cytosolic diffusion of free fatty acids and help mitigate lipotoxicity [22].

Mitochondrially associated CLDs are thought to contribute to myocardial metabolic flexibility, which is the heart’s ability to alternate between fatty acid and glucose oxidation in response to changes in nutritional status and workload. Under fasting conditions, activation of the PPARα–PGC-1α pathway is associated with increased cardiac PLIN5 expression [23], and PLIN5-decorated CLDs are thought to exhibit enhanced mitochondrial coupling and lipolytic activity, thereby supporting a greater reliance on fatty acid oxidation for energy production in cardiomyocytes. In skeletal muscle, prolonged fasting has been shown to increase PLIN5 association with LDs and to alter LD morphology [47], suggesting that similar dynamics may occur in the heart. Conversely, in the fed state or under conditions of high glucose availability, insulin signaling suppresses cardiac lipolysis and is accompanied by a greater reliance on glucose oxidation. This dynamic remodeling of the CLD pool is considered important for the heart’s ability to adapt to varying physiological conditions while preserving contractile function [23,47].

The molecular basis for the functional specialization of mitochondrially associated CLDs primarily involves the PLIN family of LD surface proteins, among which PLIN5 plays a central role. PLIN5 is highly and specifically expressed in the healthy heart and facilitates the establishment of stable membrane contact sites between CLDs and mitochondria through its C-terminal domain. The molecular basis of this tethering, specifically the interaction between the C-terminal domain of PLIN5 and FATP4, has been demonstrated in noncardiac cell lines [38]; we speculate that a similar mechanism may operate in cardiomyocytes, although direct evidence in the heart is currently lacking. This structural linkage promotes the efficient transport of fatty acids released during lipolysis to the mitochondria for β-oxidation, thereby helping to meet the fluctuating ATP requirements of rhythmic cardiac contractions. Conversely, under basal conditions, PLIN5 restricts excessive lipolysis, safely sequesters surplus fatty acids within the LD core, and reduces the production of mitochondrial reactive oxygen species, which may help protect cardiomyocytes against lipotoxicity [11,28,38,48,49].

In addition to PLIN5, PLIN2 is widely expressed in healthy cardiomyocytes, where it regulates CLD stability and basal lipid turnover. PLIN2 is thought to restrict excessive basal lipid breakdown and promote the lipophagocytic degradation of CLDs, thereby contributing to the fundamental lipid buffering capacity of the heart and limiting the accumulation of lipotoxic lipid intermediates [15,32,45,50]. Although PLIN1, PLIN3, and perilipin 4 (PLIN4, encoded by *Plin4*) are minimally expressed in cardiomyocytes under basal conditions [15], it remains unclear whether they play specific roles in defining distinct CLD subpopulations under physiological or pathological conditions.

Together, the distinct PLIN proteins present on CLDs—particularly PLIN5 and PLIN2—are thought to contribute to the functional specialization of different CLD subpopulations and may regulate lipid homeostasis, energy utilization, and metabolic flexibility in healthy hearts. Dysregulation of these PLIN proteins has been linked to various cardiac pathologies, suggesting that the PLIN-mediated functional identity of CLD subpopulations is relevant to both physiological maintenance and progression of disease.

### 4.2. Influence of Lipid Droplet Subgroups of Different Sizes on Cardiac Function

In the healthy adult myocardium, CLD size is not static and appears to vary in response to the metabolic conditions. Changes in CLD size have been associated with shifts in cardiac energy demand and lipid availability, suggesting a possible link between CLD size dynamics and the maintenance of contractile function and metabolic adaptability. Alterations in CLD size may also affect CLD–mitochondria interactions and contribute to disturbances in the lipid metabolism. Under pathological conditions, such as heart failure, the size and subcellular distribution of CLDs are altered in both human and mouse hearts [7,8]. CLD size is not only altered in disease but also dynamically shaped by physiological metabolic state: under matched fasting conditions, CLD size can differ by up to threefold across mouse genotypes with distinct metabolic profiles [7]. While the mechanisms underlying physiological CLD remodeling in the heart remain incompletely defined, studies in skeletal muscle have demonstrated that prolonged fasting selectively increases both the number (+23%) and size (+23%) of PLIN5-positive LDs [47], suggesting that PLIN5 may play a similar role in regulating CLD dynamics. Collectively, these observations suggest that CLD size is dynamically regulated in response to metabolic demands and that PLIN5 may participate in this regulation [51,52].

In the healthy adult myocardium, the size of CLDs is thought to be under tight regulatory control. Changes in CLD size are closely linked to the energy demands and lipid balance of the heart, which are important for maintaining normal contractile function and metabolic adaptability of the heart. Alterations in CLD size may impair CLD–mitochondria interactions and contribute to lipid metabolism disorders. Both pathological conditions and physiological stressors, such as fasting, have been associated with changes in CLD size, and these alterations have been linked to PLIN5 [7,8,47].

The surface proteome of CLDs is believed to influence their dynamic behavior and functional characteristics. In addition to the PLIN family, other LD-associated proteins, including lipolytic enzymes, CLD–mitochondrial tethering proteins, and small GTPase regulators, may contribute to the regulatory framework that governs the function of CLDs. Dysregulation of these proteins may impair lipid and energy homeostasis in the myocardium, potentially contributing to cardiac injuries.

Myocardial lipolysis is primarily mediated by the ATGL–abhydrolase domain containing 5 (ABHD5)–HSL axis on the CLD surface, which links lipid storage within CLDs to myocardial energy provision. ATGL, which is localized on the CLD surface, is the rate-limiting enzyme for myocardial lipolysis and catalyzes the breakdown of triglycerides within the CLD core to release free fatty acids. Its activity is regulated by PLIN5 and ABHD5. In vitro studies using COS-7 cells and isolated cardiac LDs have shown that PLIN5 restricts ATGL-mediated lipolysis under basal conditions and that PKA-mediated phosphorylation of PLIN5 at serine 155 relieves this restriction, facilitating lipid mobilization in response to increased energy demands [49,53]. Overactivation of ATGL is associated with uncontrolled myocardial lipolysis, which may result in the accumulation of free fatty acids in the cytoplasm, potentially inducing lipotoxicity and mitochondrial oxidative stress. Moreover, ATGL overactivation has been implicated in myocardial hypertrophy and heart failure. Conversely, loss of ATGL function results in impaired myocardial lipolysis, preventing the release of fatty acids for energy and promoting the excessive accumulation of CLDs in the myocardium. This accumulation is associated with severe myocardial fatty degeneration and a progressive decline in cardiac function and is considered a key pathogenic mechanism of diabetic cardiomyopathy [49,51,52,54].

ABHD5, a specific coactivator of ATGL, is an important regulator of lipolysis in the myocardium. In adipocytes and in vitro systems, ABHD5 is sequestered by PLIN1 or PLIN5 on the surface of LDs under basal conditions, limiting its interaction with ATGL. Upon β-adrenergic stimulation, PKA-mediated phosphorylation of PLIN releases ABHD5, which activates ATGL [54]. A similar mechanism involving PLIN5 and ABHD5 may operate in cardiomyocytes, as PKA-mediated phosphorylation of PLIN5 has been shown to regulate cardiac lipolysis [49,53]. However, direct evidence of ABHD5 release from PLIN5 on the CLD surface in cardiomyocytes remains limited. Impairment of ABHD5 function may disrupt myocardial lipolysis and impede ATGL activation, even under energy deficiency conditions. Consequently, this disruption is associated with the excessive accumulation of CLDs in the myocardium, which may contribute to inadequate energy provision, myocardial insulin resistance, and diminished cardiac performance. Notably, Chanarin–Dorfman syndrome, caused by loss-of-function mutations in ABHD5 in humans, manifests as severe cardiomyopathy and widespread lipid accumulation across multiple organs [54,55].

## 5. Influence of Lipid Droplet Heterogeneity on the Development of Heart Diseases

Dysfunction, mislocalization, or abnormal expression of PLIN family proteins on cardiomyocyte CLDs may impair CLD homeostasis and cardiac physiological functions, contributing to various cardiovascular and cardiometabolic diseases. PLIN1, a member of the PLIN family, is not expressed in cardiomyocytes and is therefore not discussed in detail here; however, its dysfunction in adipose tissue can indirectly affect cardiac function through systemic lipid metabolism [28,56].

PLIN2, the predominant CLD coat protein in cardiomyocytes under basal conditions, has dual pathological effects when it is dysregulated. Excessive PLIN2 expression on CLD surfaces is associated with abnormal CLD accumulation, ventricular steatosis [57], and atrial myocardial electrical remodeling with disrupted Cx43 distribution, which may increase the risk of ventricular fibrillation [32]. Conversely, inadequate PLIN2 levels have been shown to impair the lipophagic degradation of CLDs, contributing to anomalous myocardial triglyceride accumulation, which is associated with marked cardiac dysfunction and increased myocardial infarct size after myocardial infarction [50].

Proteomic analyses have revealed that PLIN3 is expressed on cardiac LDs, and studies in noncardiac cells have implicated PLIN3 in the early stages of LD biogenesis [27,58]. However, PLIN3 remains poorly characterized in the heart [28], and its association with cardiac diseases is unknown. Future studies investigating the role of PLIN3 in cardiomyocyte LD biology and its potential contribution to cardiac pathology are needed.

PLIN4 is expressed at lower levels in the heart, and studies in other cell types have suggested that it plays a role in stabilizing nascent LDs [59]. Systemic knockout of *Plin4* is associated with concurrent downregulation of myocardial PLIN5 expression, reduction in basal lipid accumulation in myocardial CLDs, and impaired adaptive regulation of myocardial energy metabolism [60]. These findings raise the possibility that PLIN4 indirectly influences myocardial lipid homeostasis. However, whether PLIN4 directly participates in CLD heterogeneity or contributes to the progression of cardiovascular disease remains unclear.

Dysregulated expression of *Plin5*, the most extensively studied gene in healthy hearts, is closely associated with severe myocardial dysfunction [28]. Deletion of PLIN5 on the surface of cardiomyocytes with CLDs impairs the formation of contact between the CLD and the mitochondrial membrane, diminishes fatty acid transport and oxidation efficiency, and may trigger uncontrolled lipolysis. Consequently, free fatty acids and toxic lipid intermediates accumulate in the cytoplasm, which may induce myocardial lipotoxicity, excessive mitochondrial reactive oxygen species production, oxidative stress, and progressive cardiac dysfunction [23]. In mouse models, deletion of *Plin5* significantly reduced the number and size of myocardial CLDs, which was associated with exacerbated myocardial damage and worsened cardiac function decline after ischemia–reperfusion injury [61]. Genetic variations in *Plin5* that lower myocardial PLIN5 levels in individuals with coronary artery disease are linked to an elevated risk of unfavorable post-ischemic consequences [61]. In diabetic cardiomyopathy, reduced PLIN5 levels are associated with attenuated diabetes-related myocardial lipid buildup, oxidative stress, and cardiac dysfunction [11]. Conversely, excessive PLIN5 expression on lipid droplet surfaces is associated with myocardial hypertrophy and impaired lipolytic enzymes, which may worsen diabetic cardiomyopathy. Aberrant overexpression of PLIN5 is associated with excessive accumulation of myocardial triglycerides and pathological cardiac hypertrophy, which may contribute to age-related decline in cardiac function [62]. Additionally, PLIN5 dysregulation has been implicated in the progression of heart failure induced by pressure overload and high-fat diets. From a systemic perspective, *Plin5* deficiency may accelerate atherosclerosis progression by contributing to endothelial inflammation, apoptosis, and oxidative stress. The expression and activity of PLIN5 in cardiomyocytes are regulated at multiple levels. At the transcriptional level, PPARα activates *Plin5* transcription, leading to increased PLIN5 protein levels, particularly in response to elevated fatty acid availability [26]. At the post-translational level, β-adrenergic stimulation activates protein kinase A (PKA), which phosphorylates PLIN5. This phosphorylation reduces the ability of PLIN5 to restrict lipolysis, thereby allowing fatty acid mobilization when energy demand increases [52]. These mechanisms may collectively shape the functional state of PLIN5-decorated CLDs in cardiomyocytes.

**Figure 2:**
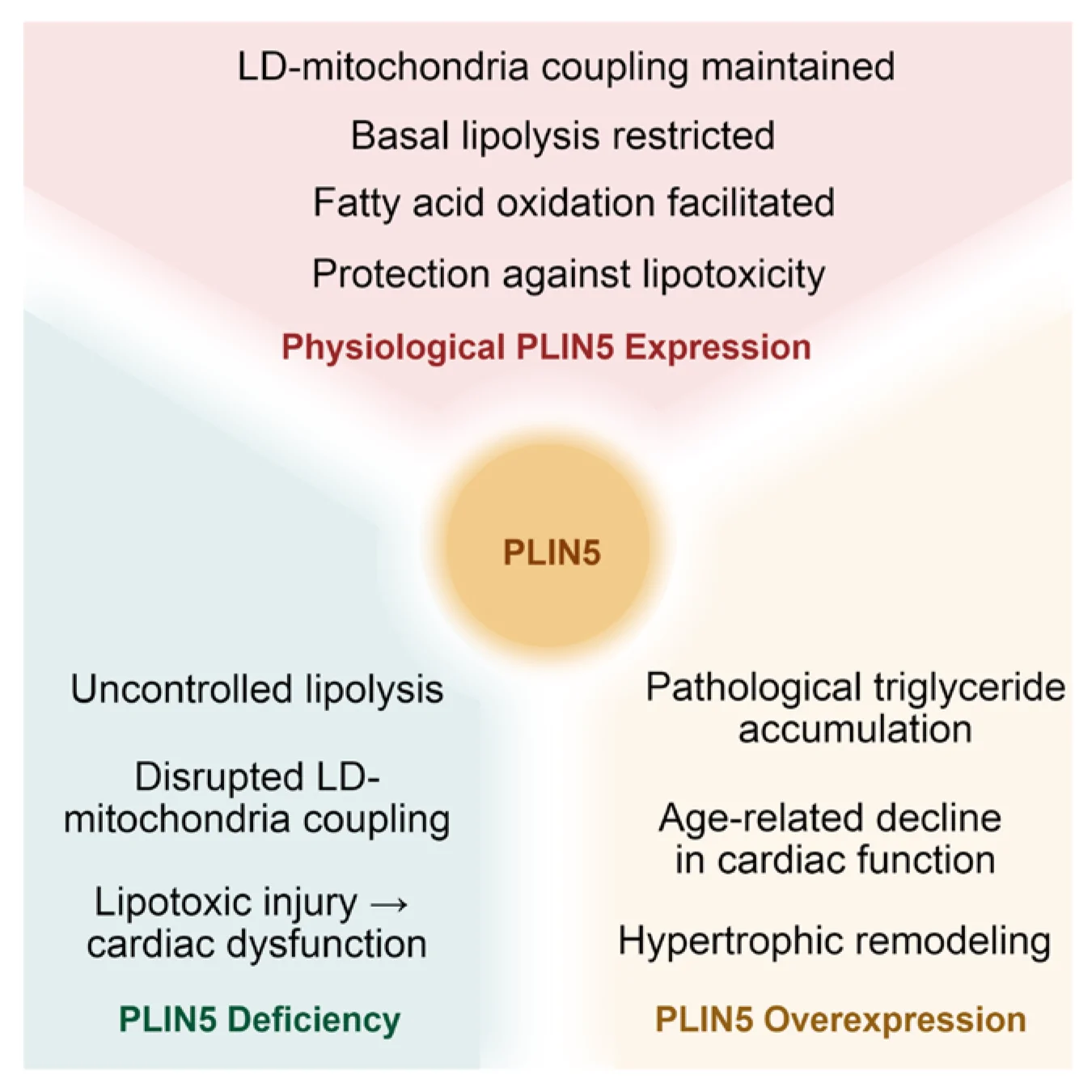
Proposed model of PLIN5 function in regulating cardiomyocyte lipid droplet biology. Schematic illustration of the proposed context-dependent roles of PLIN5 in cardiomyocytes. Under physiological expression, PLIN5 maintains LD–mitochondria coupling and restricts basal lipolysis, facilitating efficient fatty acid oxidation and protecting against lipotoxicity. When PLIN5 expression deviates from the physiological range, its protective role is lost or reversed: deficiency leads to uncontrolled lipolysis and lipotoxic injury, whereas overexpression promotes pathological triglyceride accumulation and hypertrophic remodeling. This model integrates current functional evidence and awaits further experimental validation. Created with BioGDP.com (https://BioGDP.com) [29].

Taken together, these findings suggest that the functional role of PLIN5 in cardiomyocytes is closely related to its expression level and post-translational modification status. A proposed model of PLIN5 function across its expression range is illustrated in Figure 2. Under physiological conditions, PLIN5 is thought to maintain LD–mitochondria coupling and restrict basal lipolysis, thereby facilitating efficient fatty acid oxidation while helping to protect cardiomyocytes from lipotoxicity. Under conditions of acute energy demand, PKA-mediated phosphorylation of PLIN5 is thought to regulate its lipolytic barrier function, potentially by relieving its interaction with ABHD5 and ATGL, thereby enabling a rapid increase in lipolysis to meet the elevated ATP requirements. However, when PLIN5 expression deviates from the physiological range, its protective role is either lost or reversed. *Plin5* deficiency disrupts LD–mitochondrion contact sites and triggers uncontrolled lipolysis, leading to lipotoxic injury and cardiac dysfunction. Conversely, excessive PLIN5 expression promotes pathological triglyceride accumulation and hypertrophic remodeling, which are associated with age-related decline in cardiac function. Thus, the expression and post-translational modification of PLIN5 in CLDs are important determinants of cardiac lipid homeostasis, and its dysregulation is closely associated with cardiac dysfunction. However, the precise molecular mechanisms through which PLIN5 expression and modification influence cardiac function remain unclear, and further studies are needed to clarify how PLIN5 dysfunction contributes to the progression of cardiac diseases.

Beyond PLIN family-mediated alterations in CLD heterogeneity, changes in the number and functional state of lipid droplets within cardiomyocytes can also disrupt CLD homeostasis and contribute to cardiac dysfunction. Under conditions of lipid excess, accumulation of intramyocardial lipids, as occurs in obesity and diabetes, has been associated with cardiac lipotoxicity, myocardial fibrosis, cardiomyocyte apoptosis, and reduced contractility, ultimately contributing to heart failure [63]. Conversely, when the functional reserve of CLDs is insufficient, the heart may face a different form of metabolic failure. Mice with cardiomyocyte-specific deletion of BSCL2/Seipin, a protein required for normal lipid droplet homeostasis, develop excessive ATGL-dependent lipid mobilization, resulting in near-complete loss of lipid droplets, depletion of major myocardial lipid classes, and eventual reductions in ATP content with systolic dysfunction [64]. Partial inhibition of fatty acid oxidation or increased dietary lipid supply improves cardiac function in this model, indicating that this form of contractile failure arises primarily from inadequate lipid reserves rather than from lipotoxicity [64]. In addition, reactive oxygen species (ROS) have been implicated in the progression from lipid dysregulation to cardiac remodeling: in diabetic cardiomyopathy, ROS are associated with myocardial lipid accumulation, lipotoxicity, and decreased cardiac efficiency, in part through mitochondrial uncoupling [65]. Consistent with this, cardiac-specific overexpression of PLIN2 leads to atrial steatosis and connexin 43 remodeling, which are linked to conduction disturbances and increased susceptibility to atrial fibrillation [32]. Collectively, these observations suggest that dysregulation of the cardiac CLD pool—whether through excess accumulation or functional insufficiency—contributes to cardiac steatosis, oxidative stress-related injury, and the progression toward adverse remodeling and heart failure. A comparison between the physiological defensive roles of distinct CLD states and their pathological dysregulation is provided in Table 2.

Cardiac lipid droplet metabolism is regulated by several interacting pathways that act at different levels. At the transcriptional level, PPARα establishes the baseline expression of genes required for both fatty acid oxidation and LD-associated proteins, including *Plin5* and genes involved in lipolysis. This transcriptional program determines the capacity of cardiomyocytes to form and maintain different CLD states, and is particularly important for matching long-term lipid availability to LD storage [26]. Superimposed on this baseline is an acute post-translational layer mediated by the β-adrenergic/cAMP/PKA pathway. Upon β-adrenergic stimulation, PKA phosphorylates PLIN5 at serine 155, which relieves the PLIN5-mediated restraint on ATGL and permits rapid lipolysis to meet sudden increases in energy demand [52]. In addition, AMPK functions as an energy-sensing regulator. Under conditions of ATP depletion, AMPK phosphorylates PGC-1α, which in turn cooperates with PPARα to upregulate fatty acid oxidation genes such as *Cpt1b* and *Vlcad* [66]. This AMPK–PGC-1α–PPARα axis couples energy status to fatty acid oxidation, and its suppression has been implicated in diabetic cardiomyopathy, heart failure, and atrial fibrillation [66]. Altogether, these pathways regulate the balance between LD storage, lipolytic mobilization, and fatty acid oxidation.

The findings reviewed in this chapter suggest that aberrant expression or dysfunction of PLIN proteins, as well as changes in the number and functional state of cardiac lipid droplets, can disturb myocardial lipid homeostasis, promote lipotoxicity and oxidative stress-related injury, and contribute to adverse remodeling and cardiac dysfunction. These alterations may participate in the pathogenesis of cardiovascular and cardiometabolic diseases, although the precise mechanisms by which CLD dysregulation influences disease progression remain to be fully clarified.

## 6. Therapeutic Potential of Targeting Cardiomyocyte Lipid Droplet Heterogeneity

As discussed in the preceding chapters, CLD heterogeneity is associated with myocardial dysfunction. Therefore, we propose that CLD heterogeneity may represent a potential therapeutic target for cardiovascular diseases. In concept, interventions that selectively restore the balance among specific CLD subpopulations, rather than indiscriminately reducing total myocardial lipid content, could help preserve LD–mitochondrion coupling and restore myocardial metabolic homeostasis while maintaining the fundamental physiological functions of CLDs.

Models of *Plin5* deletion and overexpression have established its importance in cardiac lipid metabolism and function [61]. Genetic variations in *Plin5* that reduce its cardiac expression are associated with impaired post-ischemic cardiac function and worsened prognosis in patients with coronary artery disease [61]. These observations suggest that therapeutic strategies aimed at enhancing PLIN5 expression or activity may be promising for cardioprotection, although the translational potential of such approaches remains to be evaluated.

Recently, lipid droplets with distinct polarities have been identified in cardiomyocytes, and their altered polarity may be linked to the onset of heart failure. To address this, Xu et al. developed a BODIPY-based probe to assess changes in the lipid droplet polarity of cardiomyocytes. This noninvasive and visual approach offers a means to monitor lipid metabolism in individuals, which may aid in the management of heart failure [67].

RTN3 is a protein predominantly expressed in the heart and localized primarily in cardiomyocytes. In a mouse model subjected to a high-fat diet, the upregulation of RTN3 expression correlated with an increase in CLD content within cardiomyocytes, accompanied by a decrease in indicators of cardiac function. Injection of adeno-associated virus 9 (AAV9) expressing RTN3 into the myocardium confirmed that RTN3 overexpression was positively associated with both the quantity and area of CLDs in cardiomyocytes. *Rtn3*-specific knockout mice fed a high-fat diet demonstrated that the absence of RTN3 could mitigate lipid accumulation in the myocardium induced by a high-fat diet. This function of RTN3 is mediated by the distribution of fatty acids. Consequently, inhibiting RTN3 expression or activity may have a significant effect on cardiac dysfunction in obese patients [21].

In vitro and in vivo studies have revealed that the formation of LDs of varying sizes may depend on the presence of distinct proteins. For example, in noncardiac cells, DGAT2-dependent LDs are relatively large and serve as reservoirs for energy storage and supply, whereas DGAT1 facilitates the formation of relatively small LDs under stressful conditions [33]. Thus, a similar mechanism may be present in the heart, suggesting that the regulation of different proteins could specifically influence distinct CLD subpopulations.

The regulation of CLD distribution in cardiomyocytes is important for cardiac lipid homeostasis. The significant involvement of the long non-coding RNA LIPTER in the transport of CLDs suggests its potential as a therapeutic avenue for heart diseases associated with the metabolic syndrome [12]. Interventions that restore or enhance the function of mitochondrial-associated CLDs have shown cardioprotective effects in preclinical models [13], suggesting a potential therapeutic strategy for improving cardiac function. However, research in this area faces significant challenges, including the lack of noninvasive, real-time methods for monitoring dynamic changes in CLD subpopulations in the myocardium. Moreover, targeting and regulating specific CLD subgroups presents a substantial practical challenge. Furthermore, the complexity of regulatory networks within organisms raises concerns regarding the potential for unknown functional impairments resulting from therapeutic manipulation of CLD heterogeneity. Although therapeutic approaches targeting CLD heterogeneity require further investigation, they represent a promising direction for future cardiovascular research.

Translating these therapeutic strategies into practice will require addressing several key issues. First, the choice of delivery system is critical to achieving cardiac-specific targeting of CLD heterogeneity in rats. Adeno-associated virus vectors, particularly cardiotropic serotypes such as AAV9, have been used to deliver PLIN5 and LIPTER in preclinical models; however, issues of dose control, tissue specificity, and long-term expression regulation remain unresolved. Second, the safety of these interventions warrants careful evaluation. Since PLIN5 overexpression promotes myocardial triglyceride accumulation and PLIN2-induced cardiac steatosis is associated with arrhythmogenic remodeling, the potential proarrhythmic effects of chronically enhanced LD formation must be assessed [32]. Similarly, the effects of these interventions on myocardial reactive oxygen species production and redox homeostasis should be monitored, given the complex relationship between lipid metabolism and oxidative stress. Finally, in first-line animal efficacy studies, primary endpoints should extend beyond reductions in total myocardial lipid content to include functional readouts of CLD heterogeneity, such as the proportion of CLDs in contact with mitochondria, rates of fatty acid oxidation, and echocardiographic measures of cardiac function. These integrated endpoints will be essential for determining whether manipulation of CLD heterogeneity confers therapeutic benefits beyond those achieved by simply lowering myocardial lipid levels.

## 7. Discussion and Future Perspectives

The heterogeneity of lipid droplets has been extensively studied in various tissues and cell types in recent years. However, a comprehensive understanding of the heterogeneity of lipid droplets in cardiomyocytes is lacking. This review categorizes distinct lipid droplet subpopulations in the myocardium and proposes that proteins located on the LD surface may play important roles in shaping LD morphology, function, and localization. Research has indicated that proteins from the PLIN family can serve as diagnostic markers for cardiomyopathy. However, investigations into the impact of lipid droplet diversity on cardiac pathological conditions and metabolic disorders, as well as their potential as therapeutic targets, are still in their infancy.

Currently, molecular markers and functional characterization of distinct cardiomyocyte-specific lipid droplet subpopulations are lacking. Future studies should integrate single-cell proteomics, spatial lipidomics, and proximity labeling approaches to comprehensively identify surface protein markers of cardiomyocyte-specific LDs, thereby elucidating the lipid composition, protein profile, and metabolic features of distinct CLD subpopulations. CRISPR-Cas9 technology can subsequently be used to generate transgenic mice expressing markers specific to individual CLD subpopulations, enabling in vivo tracking of their origin, dynamic changes, and fate under both physiological and pathological conditions. Ultimately, these efforts may lead to the establishment of a functional classification system for CLD subpopulations in the future.

Second, the interaction of CLDs with organelles such as mitochondria, the ER, and lysosomes is thought to be important for their proper functioning. However, whether distinct CLD subpopulations preferentially associate with specific organelle subpopulations is unclear. Future research should employ ultrahigh-resolution microscopy techniques, such as STED and SIM, as well as cryo-electron tomography to investigate the ultrastructure and molecular composition of CLD–organelle contact sites in cardiomyocytes. Furthermore, elucidating the specific molecular mechanisms that mediate the interactions between different CLD subpopulations and organelles and clarifying their regulatory roles in myocardial energy metabolism and lipid toxicity are essential.

Different pathological stimuli may induce distinct remodeling patterns in CLD subpopulations; however, the molecular switches that regulate this remodeling remain poorly understood. Future research should prioritize common pathological models, such as heart failure, and employ transcriptomic and proteomic analyses to identify key transcription factors and post-translational modification sites that control the conversion between CLD subpopulations. Elucidating how these molecular switches promote the conversion of CLDs into “energy storage” or “lipotoxic” subtypes—by either enhancing or suppressing the subcellular localization and activity of triglyceride synthases, including glycerol-3-phosphate acyltransferase 3 (GPAT3) and DGAT2, respectively—is essential.

Targeted regulation of CLD heterogeneity represents a promising avenue for treating cardiovascular diseases. However, subgroup-specific intervention methods are currently lacking, and achieving precise regulation of specific CLD subpopulations represents an important goal for future research. From a clinical perspective, the assessment of CLD heterogeneity in patients faces fundamental challenges, as no existing noninvasive modality can directly resolve distinct CLD subpopulations or quantify LD–mitochondria coupling in the human heart. Currently available imaging techniques, such as proton magnetic resonance spectroscopy for myocardial triglyceride quantification or PET-based assessment of fatty acid metabolism, reflect the overall myocardial lipid content and metabolic activity, but cannot capture the subcellular organization or heterogeneity of CLDs [68]. The development of cardiac-targeted imaging probes or circulating biomarkers capable of reporting the composition, distribution, or functional status of CLDs represents a transformative advance, enabling the noninvasive evaluation of CLD heterogeneity and providing a foundation for patient screening and treatment monitoring in future clinical applications [67].

Further research in the aforementioned areas could significantly advance the field; however, fundamental scientific inquiries remain. One such question pertains to whether the identity of CLD subpopulations in the myocardium is immutable or flexible. Is it feasible for distinct subpopulations to transition between each other, and if so, what mechanisms underlie this process? Addressing these questions will enhance our understanding of myocardial lipid droplet biology and offer novel theoretical frameworks and targets for intervention in cardiovascular disease prevention and treatment.

The heterogeneity of lipid droplets across multiple tissues and cell types has attracted increasing attention in recent years. A growing number of studies have revealed the diversity of LDs in the heart, highlighting the functional complexity of cardiac lipid droplets. Previous reviews have provided valuable summaries of the biology of cardiac LDs, particularly the roles of perilipin family proteins, and have discussed the significance of LD accumulation in cardiac diseases. However, these studies have primarily focused on the overall myocardial lipid content or individual LD-associated proteins. In this review, we integrate these and other findings to propose the concept of CLD heterogeneity. Drawing on existing approaches to LD classification in other cell types, we organize the observed diversity of CLDs along three dimensions—morphology, organelle connectivity, and molecular composition—and discuss how organelle contact sites may shape the functional specialization of distinct CLD subpopulations. By bringing together observations that have largely been discussed separately, this review offers a perspective for examining cardiac lipid biology through the lens of CLD heterogeneity, and we hope that it may provide a useful reference for future studies on cardiac function and disease.

This review is limited in that it focuses primarily on LD heterogeneity in cardiomyocytes and does not address other cardiac cell types, such as fibroblasts and immune cells, which also contribute to cardiac functions. Furthermore, although the diversity of LD distribution has been characterized in skeletal muscle, where LDs are categorized as intermyofibrillar or subsarcolemmal based on their subcellular localization, similar analyses are lacking for cardiomyocytes. In conclusion, this review provides a conceptual framework for understanding CLD heterogeneity and its potential relevance to cardiovascular disease and highlights key questions and future directions in this emerging field.

## 8. Conclusions

The emerging picture of cardiac lipid droplet biology challenges the traditional view that lipid droplets within a cardiomyocyte are a relatively uniform population. Rather, the heart appears to possess multiple, functionally distinct populations of lipid droplets whose specialized roles are intimately linked to their subcellular positioning and molecular decoration. This heterogeneity is not merely a descriptive curiosity: it appears to be a fundamental feature through which the heart balances efficient energy supply with safe lipid storage and metabolic flexibility.

Further work is needed to understand how this balance is maintained in health and disrupted in disease. In particular, the development of techniques capable of resolving individual lipid droplets at the molecular level, together with in vivo approaches to imaging their spatial organization, will be critical for testing whether selectively enhancing or suppressing distinct CLD subpopulations can be translated into meaningful therapeutic strategies for cardiovascular diseases.

## Figures and Tables

**Table 1:** Criteria used to assess cardiomyocyte lipid droplet heterogeneity.

Dimension	Distinguishing Criterion	Supporting Evidence
Morphology	Size and number of CLDs	Healthy: predominantly <0.5 μm; lipotoxic stress: 0.3–1.9 μm
Organelle connectivity	Preferential association with specific organelles	>70% of CLDs in direct contact with mitochondria under basal conditions; ER and lysosome associations less characterized
Molecular composition	Surface protein markers	PLIN5-enriched CLDs (mitochondria-coupled); PLIN5-reduced, HSL-increased CLDs (lipolytically active)

Note. References for the data presented in this table are provided in the main text.

**Table 2:** Physiological defense versus pathological dysregulation of CLDs.

CLD Function	Physiological Defensive Role	Pathological Alteration	Associated Molecules	Evidence Level
Energy supply	Mitochondrion-associated CLDs channel fatty acids to mitochondria for β-oxidation	Impaired LD–mitochondrion coupling; reduced fatty acid oxidation	PLIN5, MFN2	Supported by in vivo and in vitro evidence
Lipid buffering	CLDs sequester surplus fatty acids as triglycerides, limiting lipotoxicity	Excessive triglyceride accumulation	PLIN2	Supported by in vivo evidence
Lipolysis regulation	Controlled lipolysis matches fatty acid release to energy demand	Uncontrolled lipolysis; cytosolic free fatty acid accumulation	PLIN5, ATGL, ABHD5	Supported by in vivo and in vitro evidence
Lipophagy-mediated degradation	Lysosome-mediated degradation of damaged or excess CLDs	Impaired lipophagy; accumulation of damaged CLDs	PLIN2	Supported by in vivo evidence (*Plin2*-deficient mice)

## Data Availability

No new data were created or analyzed in this study. Data sharing is not applicable to this article.
